# Forensic Diatom Analysis: Where Do We Stand and What Are the Latest Diagnostic Advances?

**DOI:** 10.3390/diagnostics14202302

**Published:** 2024-10-16

**Authors:** Stefano Tambuzzi, Guendalina Gentile, Riccardo Zoia

**Affiliations:** Department of Biomedical Sciences for Health, Section of Legal Medicine, University of Milan, 20133 Milan, Italy; guendalina.gentile@unimi.it (G.G.); riccardo.zoia@unimi.it (R.Z.)

**Keywords:** diatom analysis, diagnostic techniques, diagnostic advances, forensics

## Abstract

**Background:** diatoms are unicellular algae that have been used for more than a century for forensic purposes to diagnose drowning, with more or less success depending on the historical era. Although many years have passed, scientific research on diatoms has never ceased, which testifies to their enduring allure in forensics. Of course, diatom research has evolved and expanded over time, changing with the availability of new techniques and technologies. The volume of articles and their production over a period of many years has resulted in old, current, and new knowledge on diatoms being scattered over a large number of books and articles. **Objectives**: the purpose of this narrative literature review is, therefore, to summarize all this information and bring it together in a single work that can be useful for those who are studying diatoms and their usefulness for forensics for the first time, for those who are looking for proven methods of analysis, and finally for those who are interested in exploring new frontiers of research. **Methods**: a comprehensive literature search that included all studies dealing with the applications of diatoms in forensic science was performed in the most popular electronic databases. **Results**: traditional methods have been complemented by molecular and imaging methods and, more recently, by artificial intelligence. In addition, new biological substrates have been found for the analysis of diatoms. **Conclusions**: all this has led, on the one hand, to the consolidation of a whole body of knowledge on diatoms, on which this forensic analysis is still based, and, on the other hand, has opened up numerous new research directions.

## 1. Introduction

Diatoms are unicellular eukaryotic algae that belong to the Bacyllariophiceae and are between a few micrometers and 2 mm in size. They are almost ubiquitous and colonize almost all aquatic habitats, such as rivers, lakes, ponds, and oceans. They are characterized by an enormous diversity: to date, more than 200 genera and almost 200 thousand species are known. They are characterized by a rigid cell wall consisting of silicon dioxide, known as the frustule. It consists of two overlapping valves, of which the upper one is called the epivalve and the lower one is the hypovalve, which are folded back on themselves and form a closed structure. In some cases, these two valves are connected along a median cleft called the raphe, which is responsible for movement/adhesion to surfaces [1,2,3]. Morphologically, two types are traditionally distinguished: centric, characterized by radial symmetry, and pinnate, with an elongated shape and longitudinal symmetry [4]. The pinnate diatoms are distinguished by the presence or absence of the raphe [4]. It is known from the literature that diatom species vary according to habitat and are more common in closed and stagnant waters than in the sea. Moreover, the number of diatom species varies even at the same site depending on annual and seasonal rhythms and the water’s depth and physicochemical parameters [2,3,5]. Despite this pronounced variability, the particular siliceous cell wall that characterizes diatoms gives them extreme resistance to the external environment, making them unique substrates that are extremely useful for forensic investigations in the case of bodies found in water [3].

### 1.1. Historical Background

After diatoms had been observed for the first time in 1777, it was not until 1783 that the French naturalist Lamarck was the first to formally recognize and describe their existence [6]. Many years later, in 1878, the Viennese forensic pathologist Hofman first described their presence in lung fluid and theorized about their importance and possible relevance in drowning deaths [7]. It was not until the early 1900s that forensic applications of diatoms were explored when Revenstorf detected diatoms in the lungs of a victim in 1904, solving a mysterious case of drowning [8]. In 1942, Incze succeeded in extracting and detecting diatoms in the blood and parenchymatous organs of drowning victims [9]. Tamasaka extracted them from bone marrow in 1949 [10]. In the 1960s and 1970s, Timperman performed diatom analyses on extensive case records of drowning victims, providing evidence for the validity of diatom tests for the diagnosis of drowning in forensic medicine [11]. In the following years, Ludes et al. [12,13], Lunetta et al. [14], and Pollanen et al. [15] made enormous contributions in this field. Research on diatoms has never stopped, continuing into the new millennium, and is still developing today [3].

### 1.2. Forensic Relevance

The discovery of a body in water poses a major challenge, as various scenarios arise with regard to the possible cause and manner of death (accident, suicide, homicide), which have a huge impact on investigations [16,17,18]. Indeed, it is crucial to distinguish whether the victim died by drowning or whether it is a case of submersion, where a body that has died from another cause (natural or violent) is thrown into the water, perhaps precisely to simulate drowning [19]. This differential diagnosis is complicated by the fact that, from a forensic pathology perspective, there are no pathognomonic signs of death by drowning [20,21]. The only recommendation in the literature to date is that macroscopic findings of airway foam, emphysema, and pulmonary edema together with the known circumstances can only be considered highly indicative of drowning when present in well-preserved cadavers with a post-mortem interval <48 h [22]. Moreover, if it is a body in an advanced stage of decomposition or even skeletal remains, significant macroscopic findings are completely absent [23,24]. Therefore, the diagnosis of drowning is still too often made on the basis of the exclusion criterion. It is in this scenario that the complementary diatom test can play an important role, precisely because of the diatoms’ morphological characteristics and distribution in the aquatic environment [25]. In a death by drowning, the water enters the respiratory tract by inhalation and floods the lungs. The water and the diatoms it contains passes through the lung filter into the blood and reach the peripheral organs. If, on the other hand, a dead body is immersed in water, the diatoms can passively enter the respiratory tract but cannot spread to the other organs, as there is no cardiovascular activity. It follows that finding diatoms in the internal viscera of an uninjured body can strongly support the diagnosis of drowning. In addition, diatom analysis can also provide information on the location of drowning if reference water samples are available to compare the diatom species detected in the viscera [3,14,23,25,26,27,28,29].

### 1.3. Aim of the Study

It is evident that over the years, much research has been carried out on diatoms, testing various extraction and analysis methods in different forensic contexts so that old, current, and new knowledge about diatoms is scattered over many books and articles. It was therefore considered appropriate to carry out a narrative review of the literature to summarize all this information and bring it together in a single work, highlighting, in particular, the new diagnostic advances. It may be useful for those studying diatoms and their usefulness for forensics for the first time, for those looking for proven methods of analysis, and finally for those interested in exploring new research frontiers.

## 2. Materials and Methods

A literature search that included all studies dealing with the applications of diatoms in forensic science by the 9 September 2024 was performed in the most popular electronic databases (PubMed—https://pubmed.ncbi.nlm.nih.gov, Scopus—https://www.scopus.com/search/form.uri?display=basic#basic, Medline—https://www.nlm.nih.gov/medline/medline_overview.html, Google Scholar—https://scholar.google.com, and Web of Science—https://www.webofscience.com/wos/woscc/basic-search all accessed on 9 September 2024) using the following combination of text protocols “diatoms” combined with the Boolean operator “and” followed by “forensic”, individually and randomly combined with “application” and “methods”. Starting the literature review from PubMed, the search for “diatoms and forensics” returned 282 articles. The combination “diatoms and forensics” plus “application” yielded 64 articles, and the combination “diatoms and forensics” plus “methods” yielded 190 articles. Most of these articles date from after the year 2000 (221 with the search term “diatoms and forensics”). Of the total number, all articles containing new information and expanding knowledge on the topic were considered in chronological order. Therefore, original papers and case reports that used the same extraction and analysis methods as previous studies were not included, while those that introduced new methodological variants were retained, regardless of whether the outcome was better or worse. Similarly, only items that were new compared to previous studies were derived and extrapolated from the literature reviews, and those that were repetitive were not included. The remaining databases were then also checked, with duplicates removed and only articles with new elements included. Only English-language full-text articles were considered, and dated publications were also used. Lastly, all bibliographies of the selected articles have been revised to include additional relevant articles, regardless of their language, in order to include historical sources of the first investigations on diatoms in the forensic field.

## 3. Results

### 3.1. Methodological Approach to the Diatom Analysis

#### 3.1.1. Samples to Be Collected and Analysed

**Figure 1:**
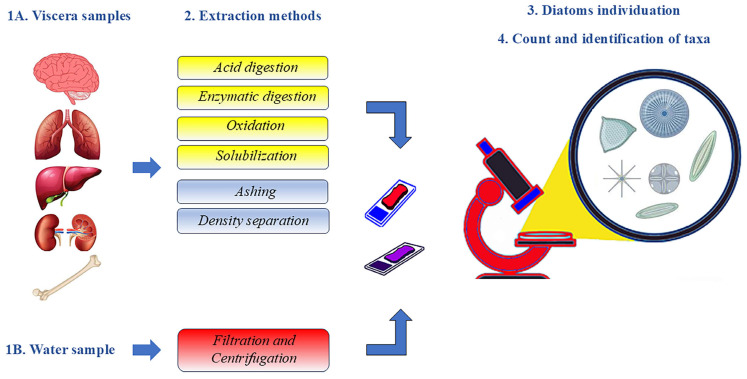
Schematic representation of the diatom analysis workflow with details of the various extraction methods known to date and described in the literature (chemical methods on a yellow background, physical methods on a grey background, and analysis of the water reference sample on a red background).

For a correct diatom analysis, both the water in which the corpse was found and specific biological samples during the autopsy must be collected [25]. The water samples should be collected from the site of the suspected drowning when the body is discovered, with samples taken from the surface and from depth in sterile containers and stored at 4 °C [25]. Regarding viscera, the literature agrees that the brain, lung, liver, kidney, and femoral bone marrow are traditionally the most suitable substrates for examination (Figure 1). In particular, bone marrow is considered the most reliable substrate for diatom analysis because it is less exposed to contamination while the body is in water. After exposing the organ to be sampled, a “wedge-shaped” fragment weighing at least 10 g must be removed.

Suppose the collected samples cannot be analyzed immediately. In that case, they should be frozen or possibly fixed in formalin, as it has been shown that diatoms can be identified even in the latter case [30,31,32].

#### 3.1.2. Centrifugation and Filtration

To separate diatoms from the water samples, it is considered suitable to centrifuge the sample for 15 min at 2500 rpm, remove the supernatant, and use a membrane filter with a pore size of 0.45 μm or 1.0 μm, which has the property of retaining diatoms and allowing other residues and substances to pass through [3]. The use of nitrocellulose filters with a pore size of 5 μm is recommended for samples with a low diatom content. Recently, a new membrane filtration method for the enrichment of diatoms in samples was proposed in the literature, in which the membrane was made transparent by a solution with different ratios of acetic acid and eugenol [33]. This method has been shown to be superior to the conventional approach. In general, acid treatment or incineration of filters is required to proceed with light microscope analysis. The digested filter residue must then be diluted with distilled water, and the dilution must be re-filtered through an additional 25-mm diameter membrane filter, which, after being left to dry, can be observed under the microscope [32,34]. Unsuitable filters can lead to the deposition of other materials, as these can clog the pores and mask the diatoms [3,35]. Alternatively, the water samples can be processed after centrifugation in a similar manner to the viscera discussed in the next section (Figure 1).

#### 3.1.3. Extraction Methods

For the extraction of diatoms from the viscera, it is considered appropriate to examine a quantity of 10 g for each organ to be investigated [3]. Various extraction methods can be used to extract and obtain diatoms [3,35,36,37]:

- Acid digestion method (chemical method):

Due to the simplicity of the process and the low cost, acid digestion is the technique that has been used since the first experiments and is still the most commonly used and generally accepted today. The sample is treated with acids to dissolve the organic substances. Pollanen, in 1997 [38], was one of the first pioneers who proposed a structured version of this method, taking inspiration from Ludes, 1994 [12], which was later followed by several variants using nitric acid, hydrochloric acid, sulphuric acid or mixtures of these acids [39,40,41,42]. A particularly effective mixture consists of 25 mL of 98% sulphuric acid and 25 mL of 69% nitric acid (generally between 60–70%) [25,43]. In addition, K_2_Cr_2_O_7_ granules can be added to accelerate the process by additional heating for three hours [32,44]. For the sake of completeness, it should be mentioned that hydrochloric acid and nitric acid have been combined with hydrogen peroxide, and in both cases, good results have been obtained [12,45]. More recently, the use of nitric acid heated to 400 °C [46] and the combination of sulphuric acid (H_2_O_4_) with oxalic acid (C_2_H_2_O_4_) and potassium permanganate (KMnO_4_) have also been tested on soft tissue, teeth and bones of rats with excellent results [47]. Finally, fuming nitric acid was combined with the use of dodecyl sulfate (SDS), a strong anionic detergent, on a cadaveric blood sample, but this was a single application with no more recent reports [34].

- Enzymatic digestion method (chemical method):

In this method, a buffer solution containing Tris-HCl (pH 8.0), EDTA, and sodium chloride is prepared and added to the sample to maintain the ionic strength and pH [12]. The enzyme proteinase-K (0.1–1 mg/mL) is then added, and the sample is incubated for several hours or overnight. A protease inhibitor is added to block the enzyme’s activity, and then the treated sample is centrifuged to isolate the diatoms [12]. Although some reports [48,49,50] have demonstrated greater efficacy of proteinase K than conventional acid digestion, this technique is too expensive for large samples. In addition, it has been observed that incomplete digestion may occur, with organic residues interfering with microscopic observations, and sensitivity may be lower in solid viscera other than lungs [51]. For this reason, this technique could be used more efficiently in the treatment of samples at an advanced stage of decomposition, as suggested by Ludes himself [12]. To improve the results, some studies recommended completing the digestion with hydrochloric acid or hydrogen peroxide to achieve complete dissolution of the residual organic matrix [30,31,51]. In 2019, Kakizaki et al. suggested that the digestion efficiency of papain (enzyme hydrolase) is similar to or better than that of proteinase K [52]. This reagent is significantly cheaper than proteinase K and reduces experimental costs by about 6-fold, although it still deserves further investigation. Over the years, the use of proteinase K has also been associated with sodium dodecyl sulfate (SDS), with no particular evidence of increased efficacy [30,31,50].

- Oxidation methods (chemical method):

The sample is treated with strong oxidizing agents such as hydrogen peroxide (H_2_O_2_) [3]. Recently, Marezza et al. have shown that the technique based on 70 °C hot hydrogen peroxide is even more effective in terms of reducing the amount of final sediment, extracting the material, and preserving the diatoms, thus proving to be a viable alternative to the traditional alternatives using acids, both in terms of cost and operator safety [53]. Finally, the use of a mixture of hydrogen peroxide and sulphuric acid (H_2_SO_4_) is also reported in the literature, in which the introduction of a highly acidic medium enhances the effect of decomposition of the organic material while preserving the diatoms [39].

- Solubilization methods (chemical method):

Occasional and not recent reports [50,54,55,56,57] have described the use of Soluene-350, which is a tissue solubilizer consisting of a strong organic base formulated with toluene. In this method, the sample must be cut into small portions and homogenized in distilled water. After centrifugation at 18,000 rpm for 10 min, the precipitate is suspended in 8 volumes of Soluene-350 and incubated at 50 °C for 2 h and then at room temperature overnight. The precipitate is ready for analysis after centrifugation at 3000 rpm for 60 min. This technique has been shown to be effective for tissue dissolution and diatom analysis, although one report found results suggesting greater preservation of freshwater diatoms than saltwater diatoms [55]. In any case, no further studies have investigated this specific aspect.

- Ashing method (physical method):

For fat-rich materials, incineration may be performed in a muffle furnace capable of maintaining temperatures of 500–600 °C for several hours. The analysis protocol proposed by Ludes [12] consists of 80 °C for 2 h, 200 °C for 8 h, and 550 °C for 2 h. Inorganics, water, and volatiles are vaporized while the organics are burned in the presence of oxygen and converted to CO_2_ and nitrogen oxides. The ash can then be treated with oxidizing agents or by acid digestion [12], followed by extraction of the diatoms [3].

- Density separation method (physical method):

This extraction technique utilizes the different density properties of diatoms and other particles in the sample by using a density gradient medium such as sodium polytungstate or Ludox (colloidal silica in water). Diatoms can also be separated from other materials based on their buoyancy or sedimentation rate [3].

The above methods are schematically included in Figure 1.

For all the chemical methods described, it is advisable to allow the samples to rest for 24–48 h (and to cool them in the case of acid digestion) before isolating the diatoms from the extracted tissue suspensions. They are then transferred to graduated tubes and centrifuged at 3500 rpm for 30 min. The supernatant is then aspirated without touching the pellet at the bottom. Samples can be re-centrifuged up to four times, adding distilled water as a replacement for the substance used to digest the sample. From the last sample, a series of slides can be mounted by filtration or cytocentrifugation until the sample is exhausted, and then they should be allowed to dry [32,35]. In the case of filtration, the precautions mentioned in the previous paragraph must be observed.

For the sake of completeness, it should be pointed out that items of clothing from victims found in the water have also been examined in the literature as possible sites for conducting diatom analyses to identify the site of drowning. In detail, Uitdehaag et al. [58] compared the dissolution method, the ethanol rinse method, and the water rinse method, with the first two being the most recommended. The transfer and persistence of diatoms in common shoe materials were also successfully investigated by Levin et al. using heated hydrogen peroxide [59].

Only recently, a method for extracting diatoms from larvae colonizing cadavers of drowned piglets was described in the literature for the first time. This method consisted of acid digestion based on K_2_Cr_2_O_7_ and H_2_O_2_, followed by traditional centrifugation and preparation of histological slides [44].

### 3.2. Diatom Evaluation

Before evaluation, it must be taken into account that diatoms are transparent and have a refractive index (r.i.) of 1.44, which is similar to that of glass. Therefore, mounts based on synthetic resins with a high refractive index must be used (e.g., naphrax, r.i. = 1.74). The light microscopic examination should be carried out at a magnification of 630–1000×, although the diatoms can also be examined with a dark-field or phase-contrast microscope. All microscopic preparations should be carefully observed, proceeding in strict order so that no field is overlooked. The number of diatoms found must be counted (density), determining the total number of diatoms per gram of tissue or organ [32,39]. Subsequently, the observed diatoms must be measured (morphometry) and recognized (species identification). It should be noted that this must be carried out for intact diatoms and fragments of diatoms that may vary in size [60]. Much has been written in the literature over the years about the significance of diatoms in cadaver samples. However, there are no standardized protocols for the quantitative and qualitative analysis of diatoms, so comparing different studies is extremely difficult. The diatom content of the water, the tissue sample studied, and the extraction procedures are all variables that can influence the results of the research. Most studies often have only empirical significance: older studies report only positivity or negativity [61], while other recent studies lack the prerequisites for quantitative analysis [39]. Different evidence regarding the significance of diatoms has been observed between the lung and the other viscera.

### 3.3. Significance of Diatoms in the Lung

This is of crucial importance as it is demonstrated that diatoms can enter the lungs of a living drowning victim by inhalation but also passively during post-mortem submersion. For this reason, this has been intensively discussed in the literature, and there has even been contradictory evidence (Table 1). For example, Tomonaga [62] demonstrated the presence of numerous diatoms in the lungs of corpses immersed in 23 m of water for 30 min. On the other hand, Nanikawa and Kotoku [63] found up to 145 diatoms/g in the lungs of corpses kept under water at a depth of 120 m for 2–3 months. Reh [64] considered the presence of diatoms in the lungs to be an unspecific finding, and Neidhart and Greendyke [65] stated that the detection of diatoms in the lungs has no diagnostic value in determining whether the victim was alive prior to submersion. In contrast, Timperman [11] argued that the presence of diatoms in the lungs is an important indication for the diagnosis of drowning, as he observed that their number does not exceed 10 frustules per 100 g in non-drowned persons. According to Ludes and Coste [66], the presence of diatoms only in the lungs but not in the internal organs does not exclude the possibility of passive penetration. According to some histological studies, however, the distribution of diatoms is different during drowning and post-mortem submersion. In the first case, the diatoms can penetrate into the alveoli of the subpleural regions; in post-mortem submersion, the diatoms may be found in the interlobular and intralobular bronchi but not in the bronchioles and alveoli [67]. At the end of the 1980s, Auer and Mottonen [68] were of the opinion that the diagnosis of drowning could be made if more than 20 diatoms per microscopic sample were found in the lung tissue. In fact, this interpretation key has been consolidated over time, as even Ludes proposed a threshold of 20 diatoms per slide from a 100 mL sediment pellet as a reliable criterion for the diagnosis of drowning [12]. This was later taken up by Lunetta et al. [69] and Fucci et al. [70].

To this day, the detection of 20 diatom frustules per 100 mL of the residue obtained from the digestion of 10 g of the lung is considered an indication of drowning before death. This was also linked to the criterion of qualitative and quantitative correspondence between the extracted diatoms and those in the reference water sample in order to avoid false positive results [32]. Overall, then, the finding of diatoms in the lung can be technical evidence of great forensic utility, especially when large numbers are detected, but it should be contextualized with the analysis of the other viscera.

### 3.4. Significance of Diatoms in the Other Viscera

Over the years, numerous studies have also been carried out on this aspect, dealing with both the presence or absence of diatoms and their quantification, although the results here also vary greatly (Table 2).

In the serial examination of 40 drowning victims carried out by Ludes and Coste, 14 were positive for the presence of diatoms in the lungs, 11 were positive in the lungs and internal organs (liver, kidney, and brain), while 15 were completely negative [66]. Auer and Mottonen [68] found diatoms only in the lungs in 33 cases, diatoms in the lungs and the internal organs in 62 cases, and completely negative in 12 cases in 107 victims who had presumably drowned. Foged [71] reported 6 to 221 valves/g in the lung, 5 to 68/g in the liver, and 9 to 127/g in the kidney, while Giri et al. [72] reported 40 diatoms/10 g in the lung, 25 in the kidney, 20 in the liver and 10 in the brain. Hurlimann et al. [60] estimated the maximum diatom density to be 54–108 diatoms/5 g in the lung, 92–184 in the liver, and 22–44 in the kidney. Ludes and Coste [66] found more than 60 diatoms/10 g in the lungs of 66% of 40 drowned victims, while the maximum content in the other organs (kidney, liver, and brain) was 15 diatoms/10 g tissue.

Only a few authors have established a quantitative threshold for diatoms that allows a diagnosis of drowning. Ludes et al. [13] proposed a threshold of 5 diatoms per histologic preparation per 100 µL of peripheral viscera as a reliable criterion for the diagnosis of drowning. Hurlimann et al. [60] suggested higher values of up to 20–40 diatoms/5 g in the bone marrow. Again, the suggestion of Ludes et al. has been consolidated over time so that the detection of at least 5 diatoms per 10 g of sediment from viscera other than the lung is considered a reliable criterion for the diagnosis of drowning. These findings were later confirmed by Lunetta et al. [69] and Fucci et al. [70]. Later, the criterion of qualitative and quantitative correspondence between the extracted diatoms and those in the reference water sample was added to avoid false-positive results [32].

### 3.5. Diatom Species Identification

This is the most complicated aspect, as it depends entirely on the expertise of the observer. Therefore, each diatom must be carefully observed to record its characteristics in terms of shape, size, and structure so that even the smallest differences that make it possible to distinguish the different species can be registered. This is an extremely time-consuming and laborious task that requires professional expertise.

Therefore, a multidisciplinary approach is essential in forensics, where a natural scientist or biologist with expertise in diatomology can assist the forensic pathologist in formulating the correct diagnosis. Despite all this, the correct classification of diatoms can sometimes be a challenge, even for experts in the field [3,32].

### 3.6. Sources of Biases in Diatom Analysis

At this point, the entire diatom analysis procedure is exposed to a high risk of contamination with several possible sources of error. This applies from organ sampling to the preparation of samples on histological slides and their evaluation in order to avoid false positive results. Therefore, it is crucial to use a strict procedural protocol.

During autopsy, it is important to proceed cautiously to minimize the risk of contamination, starting with properly handling wet clothes worn on the victim. A scalpel (or similar blade) and sterile forceps must be available before sampling each viscera. After exposing the organs to be sampled, their surface should be rinsed with highly purified (penta-distilled) water to remove any drops of contaminating water. The taken samples should be placed in a sterile container, or they must have been pretreated for 24 h in a sodium hydroxide detergent and rinsed with ultrapure water. Another precaution in bone marrow sampling is that a sterile oscillating saw blade must be used to cut the femoral diaphysis [3,15,35,36,51,73,74].

Care should also be taken in the later stages to ensure that containers are properly sterilized and rinsed with ultrapure (penta-distilled) water. Old containers are contraindicated as they may have glass irregularities in which contaminating diatoms may be present and persist. In addition, all reagents used in laboratory procedures should be regularly tested for environmental diatom contamination. Finally, paper materials, such as laboratory filters, should be avoided due to their high contaminating power.

However, the possible biases in the analysis of diatoms do not end with the laboratory procedures, as the observation and interpretation phase under the microscope can also be a source of error. For example, an observer may mistakenly fail to recognize some diatoms, especially if they are fragmented, or mistake foreign material such as pollen or other aquatic contaminants for diatoms. All of these lead to biases in the analysis of diatoms that can influence the final diagnosis [3,15,35,36,51,73,74]. Given these potential sources of error, it is also possible that different observers come to more or less different conclusions, which has a negative impact on inter-investigator reliability. To date, however, no studies have specifically investigated this aspect.

### 3.7. New Diagnostic Advances

#### 3.7.1. Innovative Biological Substrates

On the side of biological substrates on which diatoms can be analyzed for pathological-forensic purposes, there has been no innovation for decades, as research has mainly focused on the introduction of new techniques applied to traditional cadaveric substrates. This was the case until 2022, when it was pointed out that this is an unjustly neglected area of research, as there may be additional and unexplored body areas in the human body that could be of interest for diatom analysis. In particular, the report analyzed the vitreous body of cadavers and showed for the first time that it is a substrate that behaves similarly to conventional viscera in terms of diatom distribution in drowning victims. Indeed, a qualitative and quantitative correspondence was demonstrated between diatoms in vitreous humor, conventional viscera, and drowning water. These results are consistent with the fact that the vitreous humor is in a protected environment, isolated from the outside world, which is why it has been the subject of other forensic applications in post-mortem toxicology and biochemistry. This makes the vitreous humor a substrate that can be sampled and analyzed like other conventional viscera when investigating a suspected drowning death. In addition, such a substrate has the great advantage that it can be analyzed quickly and inexpensively without lengthy preparation. It is sufficient to centrifuge the vitreous sample (1 mL) at 3500 rpm for 5 min, remove the supernatant, and spot the residue onto glass slides using a cytocentrifuge at 1000 rpm for 8 min. The preparation can be stained with H&E to counteract the background or left unstained and fixed with a medium with a high refractive index prior to light microscopic analysis [75]. More recently, it has been shown that synovial fluid can also be a potentially useful substrate for diatom analysis and deserves more attention [76]. Although further investigation is of course required, it seems clear that there may be other cadaveric substrates for diatom analysis that would be worth exploring.

#### 3.7.2. Analytical/Identification Techniques

- Microwave digestion-Vacuum filtration-Automated scanning electron microscopy (MD-VF-Auto SEM)

Zhao et al. [77,78,79,80] developed a new method called the Microwave Digestion-Vacuum Filtration-Automated Scanning Electron Microscopy (MD-VFAutoSEM) by integrating and improving the traditional methods for diatom analysis. In this method, microwave digestion and vacuum filtration replace acid digestion and centrifugation. The strength of this method is the auto-SEM, with which diatoms can be easily and effectively identified. The SEM system automatically scans the filtration fields from the vacuum and creates images of the fields for qualitative and quantitative analysis. Compared to conventional methods, the MD-VF auto-SEM method saves time and allows a larger number of diatoms to be analyzed, although it is available only in a few laboratories. In addition, this method avoids the loss of diatoms that occurs with conventional centrifugation and observation under the light microscope in the laboratory [81,82]. In 2017, this technique was applied to 128 drowning cases [80] and detected diatoms in 100 percent of lung tissue samples and 97 percent of samples from the other organs (such as liver or kidney). To improve the accuracy of the test, it was also recommended to evaluate the ratio of the number of diatoms in the lung to that of the drowning fluid sample (L/D), with a ratio > 2 considered highly indicative of a cause of death by drowning [32,80]. The excellent efficacy of this method has also been demonstrated more recently by Liu et al. [83], Zhang et al. [84], Hagen et al. [29] and Wu et al. [85]. These results showed that the MD-VF-Auto SEM method can effectively improve the diatom detection rate in drowning cases.

- Molecular approaches

These methods use DNA-based procedures to identify and differentiate diatom species. They are gradually growing in popularity as they are very sensitive, accurate, and faster than traditional methods (i.e., direct observation of morphology), which are difficult and require a lot of experience and time. The molecular methods used in diatom analysis are:

- Species DNA barcoding: this method involves sequencing a short stretch of DNA from a standardized region of the genome to identify and distinguish species. The only DNA barcoding marker used for diatoms is the ribosomal DNA (rDNA) region, specifically the small subunit (SSU) or 18S rDNA gene. This genetic region is highly conserved in diatoms but contains variable regions that are useful for distinguishing different species. DNA is extracted from diatom samples and amplified using the polymerase chain reaction (PCR) to perform this method. The amplified DNA is then sequenced and compared with existing reference databases to identify the species. However, it should be noted that some diatom species have similar or identical DNA codes, making it difficult to identify species using this technique alone [3,35,36,86,87,88,89].
- Next Generation Sequencing (NGS): this method allows a very large number of short DNA sequences (between 50 and 600 base pairs) to be sequenced in a single run. These short sequences are then aligned to a reference genome or assembled de novo to reconstruct the original genetic sequences. In addition, NGS platforms can be used to perform whole genome synthesis (WGS), RNA sequencing, exome sequencing, nucleotide sequencing, and epigenetic analysis [3,35,36,86].
- Fluorescence in situ hybridization (FISH) is a molecular technique in which special nucleotide probes (tags) labeled with a fluorescent dye are used to identify a specific complementary nucleic acid sequence to be visualized. The probes are added to the diatom samples and incubated under controlled conditions to bind to their target sequences. Excess and unbound probes can be washed away, and the prepared sample can be visualized under a fluorescence microscope [3,35,36].
- DNA metabarcoding is a specialized molecular technique used to identify and interpret multiple species present in an environment by analyzing complex mixtures such as water. Metabarcoding involves sequencing complex samples in parallel using high-throughput sequencing, which allows the simultaneous identification of many taxa in the same sample. It is, therefore, fast and efficient for studying samples with high biodiversity [3,35,36].
  However, all these molecular methods are still only used to a limited extent in forensics due to the high cost, complex sample preparation, and extensive post-analytical data processing [3,35,36].

- Imaging approaches

- Nuclear Magnetic Resonance (NMR) and Inductively Coupled Plasma Hyphenated Technologies: currently, 2D and 3D NMR instrumentation techniques have proven successful in a variety of matrices and sample types and can be used to identify and classify unknown species and genera of diatoms. By coupling NMR with liquid chromatography (LC) and mass spectrometry (MS), i.e., hyphenated techniques using selective pre-analytical preparation of matrices, diatom fractions can also be classified [35].
- Atomic Force Microscopy (AFM): with this technique, it is possible to achieve a very high resolution of a solid sample, which can be visualized and measured even when it is in water or another liquid medium. In the specific case of diatoms, morphological details can be made visible with nanometer resolution that would otherwise be unrecognizable. The technique also has the advantage that the sample can be scanned vertically and horizontally. The ease of sample preparation, high sensitivity, and flexibility of different samples make AFM an excellent tool for the detection of diatoms [35,90]. For example, Almqvist et al. used this technique to study the silicate shell of the diatom Naviculapelliculosa (Bréb.) Hilse [91].

#### 3.7.3. Interpretation Techniques

Since conventional methods for identifying diatoms are time-consuming and labor-intensive and rely on manual microscopic examinations [92], the potential of automated systems for identifying and classifying diatoms has been explored for several decades. The Automatic Diatom Identification and Classification (ADIAC) project was launched in 1998 by the Vision Laboratory of the University of the Algarve. It aims to develop image databases and apply image processing and pattern recognition tools for the automatic computer-aided identification of diatoms [35]. Although this is a branch of research that dates back to the late 20th century, only more recently have advances in computer vision and image analysis led to the development of automated methods that use computer algorithms and machine learning to identify and classify diatoms [3,36]. In this context, the simplest procedural approach described in the literature involves three steps: (i) creating a database of diatom images from different views and angles using a microscope equipped with a digital camera or other image acquisition system; (ii) optimizing the captured images through operations such as noise reduction, resizing, and contrast enhancement; and (iii) extracting morphological and morphometric parameters from the images that are suitable for characterizing the diatoms [36]. At this point, the literature describes how the implementation of deep learning algorithms has made a decisive contribution [93]. In particular, the best results have been obtained with convolutional neural networks (CNN), which provide satisfactory results in image recognition and whose performance can be further improved by increasing the number of training patterns provided or by using a special model called transfer learning [94]. In 2017, Pedraza et al. conducted a study on the classification of diatom species using CNNs [95], in which images of 80 diatom species were successfully classified, and an accuracy of 99% was achieved. In 2019, Zhou et al. [96] showed that deep learning using CNNs can realize automatic inspection of diatoms from digitized scanner images of digested tissue smears. In this study, the identification model achieved an accuracy of 97.67% and better performance than human experts, with a rate/minute more than twice as high. Further studies were conducted in 2018 by Yu et al. [97] and Zhu et al. [98]. Following the study by Yu et al. [99], Deng et al. [100], and Zhang et al. [101], Turnois et al. [102] delved deeper into the topic in 2023 and proposed a new pattern evolution method for the detection and identification of diatoms in highly complex backgrounds from light microscopy photos. This new method was based on sequential transfer learning of object recognition patterns and provided better results than conventional learning and transfer. In particular, the technique enabled the construction of neural networks capable of identifying up to 29 genera or 52 species of diatoms commonly found in forensic cases.

It seems clear, then, that the advancement of artificial intelligence-based systems that can automatically identify and classify diatoms has the potential to significantly improve the scientific rigor and objectivity of forensic diatom analysis in the forensic field.

## 4. Conclusions

The traditional methodological approach is still an indispensable pillar in forensic investigations, capable of providing evidence whose validity has been confirmed many times in the literature. However, precisely because it is a topic of great forensic interest, research has repeatedly produced new methodological variants based on traditional approaches with excellent results. However, some limitations, such as the duration of the procedures and the difficulty of interpretation, remain and urge further research. In the meantime, molecular methods for identifying diatom species have appeared on the stage, which seem much more efficient and accurate than previous methods based solely on direct observation and comparison. Among the different methods, DNA coding seems to be the future of molecular identification of diatoms, as there are already several DNA databases for diatoms, and the general protocol of this method is standardized. The integration of molecular techniques with traditional morphological identification methods can certainly make great progress in the future. The combination of diatom analysis with different forensic methods inevitably requires a multidisciplinary approach that strengthens investigative capacities and allows for a more thorough forensic evaluation.

At the same time, artificial intelligence using deep learning to automatically identify and classify diatoms has recently become increasingly developed and established. There are still few studies in this regard, but the initial results are very promising and suggest that such an approach could revolutionize the forensic analysis of diatoms in the future by providing greater efficiency, accuracy, speed, and scientific rigor. Of course, it is unthinkable that human control can be disregarded, and above all, the probative value of the results provided by artificial intelligence must be carefully evaluated in court.

Finally, the continuous improvement of diatom extraction techniques and analytical methods must not lead to the neglect of research aimed at identifying new biological cadaver substrates for diatom analysis. In this regard, the recent example of the usefulness of vitreous humor for the forensic diagnosis of drowning should be an encouragement for further studies.

Therefore, it is clear that forensic diatomology is a discipline that evolves and expands over time, changing according to the new techniques and technologies that are used. In this sense, it is necessary to allow for change and innovation without losing sight of the origins of this discipline and its objectives so that it can continue to contribute to solving forensic cases.

## Figures and Tables

**Table 1:** Summary of the literature evidence about diatoms in the lungs.

Reference	Samples Analysed	Detected Diatoms	Forensic Relevance
Holden et al., 1955 [67]	Lungs of drowned rabbits vs. rabbits immersed in water after death	Diatoms in drowned rabbits were also found in bronchioles and alveoli	Useful for diagnosis of drowning
Tomonaga, 1960 [62]	Lungs of animals immersed in water after death	Numerous	Lungs can be passively reached by water in the post-mortem period
Neidhart et al., 1967 [65]	Human lungs of drowned people vs. non-drowned and not immersed subjects	Diatoms were present only in the lungs of drowned people	Useful for diagnosis of drowning
Reh, 1968 [64]	Human lungs of non-drowned people and the lungs of drowned animals	Diatoms were also found in non-drowned subjects	The diatom analysis may have some limits in forensics
Timperman, 1972 [11]	Human and animal lungs of drowned subjects vs. non-drowned subjects	<10/100 g in non-drowned subjects	A cut-off was proposed for the diagnosis of drowning
Nanikawa et al., 1974 [63]	Human lungs of a post-mortem immersed body	up to 145/g	Lungs can be passively reached by water
Auer et al., 1988 [68]	Human lungs of drowned people vs. non-drowned and not immersed subjects	Diatoms were present only in the lungs of drowned people	Useful for diagnosis of drowning
Ludes et al., 1994 [12]	Human lungs of drowned subjects vs. non-drowned and not immersed subjects	>20/100 mL in drowned subjects	A cut-off was proposed for the diagnosis of drowning
Ludes et al., 1996 [66]	Human lungs of drowned subjects vs. non-drowned and not immersed subjects	>60 diatoms/10 g in the lungs of 66% of drowned victims	Lungs are useful for the diagnosis of drowning, but the other viscera should also be tested
Lunetta et al., 2013 [69]	Human lungs of non-drowned subjects vs. human lungs pre-contaminated by diatoms post-mortem	Some diatoms were found in the lungs of non-drowned subjects, but<20/100 mL	Be careful for false positive results but still useful for diagnosis of drowning
Fucci et al., 2017 [70]	Lungs of accidentally drowned animals vs. non-drowned animals	>20/100 mL in drowned animals	Useful for the diagnosis of drowning also in forensic veterinary
Girela-Lopez et al., 2020 [32]	Literature review	>20/100 mL from 10 g of lungs	Diagnostic for drowning + qualitative and quantitative correspondence with the reference water sample

**Table 2:** Summarize of literature evidence about diatoms in the other viscera.

Reference	Samples Analysed	Detected Diatoms	Forensic Relevance
Foged, 1983 [71]	Human viscera of drowned and non-drowned subjects	5 to 68/g in the liver and 9 to 127/g in the kidney both in the drowned and non-drowned victims	Poor utility in diagnosing drowning
Auer et al., 1988 [68]	Human viscera of drowned people vs. non-drowned and not immersed subjects	Presence of diatoms only in the viscera of drowned subjects	Useful for diagnosis of drowning
Giri et al., 1993 [72]	Human viscera of drowned people vs. non-drowned and not immersed subjects	In drowned subjects > 25 diatoms/10 g in the kidney, 20 in the liver and 10 in the brain	Useful for diagnosis of drowning
Ludes et al., 1996 [66]	Human viscera of drowned people vs. non-drowned and not immersed subjects	Maximum content in the viscera other than lungs in drowned subjects was 15 diatoms/10 g tissue	Useful for diagnosis of drowning
Ludes et al., 1999 [13]	Human viscera of drowned people	Threshold of 5 diatoms per histologic preparation per 100 mL of peripheral viscera	Reliable criterion for the diagnosis of drowning
Hurlimann et al., 2000 [60]	Human viscera of drowned subjects	Values > 20–40 diatoms/5 g in the bone marrow in drowned subjects	Reliable criterion for the diagnosis of drowning
Lunetta et al., 2013 [69]	Human viscera of non-drowned subjects vs. human lungs pre-contaminated by diatoms post-mortem	Diatoms were found only in the viscera of non-drowned subjects, <5/10 g of tissue	Be careful for false positive results, but still useful for diagnosis of drowning
Fucci et al., 2017 [70]	Viscera of accidentally drowned animals vs. non-drowned animals	>5/10 g of tissue in drowned animals	Useful for the diagnosis of drowning also in forensic veterinary
Girela-Lopez et al., 2020 [32]	Literature review	>5/100 mL from 10 g of tissue	Diagnostic for drowning + qualitative and quantitative correspondence with the reference water sample
